# Polarization-Sensitive Surface-Enhanced In Situ Photoluminescence Spectroscopy of *S. aureus* Bacteria on Gold Nanospikes

**DOI:** 10.3390/s20092466

**Published:** 2020-04-27

**Authors:** Irina Saraeva, Sergey I. Kudryashov, Pavel Danilov, Nikolay Busleev, Eteri R. Tolordava, Andrey A. Rudenko, Dmitriy Zayarny, Andrey Ionin, Yulia M. Romanova

**Affiliations:** 1 P. N. Lebedev Physics Institute of Russian Academy of Sciences, Leninskiy Prospect 53, 119991 Moscow, Russia; kudryashovsi@lebedev.ru (S.I.K.); pavel-danilov2009@yandex.ru (P.D.); busleevni@lebedev.ru (N.B.); tolordava.eteri@yandex.ru (E.R.T.); aa_rudenko@mail.ru (A.A.R.); zayarniyda@lebedev.ru (D.Z.); andrey.ionin@gmail.com (A.I.); 2 Faculty of Photonics, ITMO University, Kronverkskiy Prospect 49, 197101 Saint-Petersburg, Russia; 3 N.F. Gamaleya Federal Research Centre of Epidemiology and Microbiology, Gamalei Street 18, 123098 Moscow, Russia; genes2007@yandex.ru

**Keywords:** nanospikes, gold, sensorics, Raman spectroscopy, antibacterial

## Abstract

We report the possibility of a time-resolved bacterial live/dead dynamics observation with the use of plasmonic nanospikes. Sharp nanospikes, fabricated on a 500-nm thick gold film by laser ablation with the use of 1030-nm femtosecond pulses, were tested as potential elements for antibacterial surfaces and plasmonic luminescence sensors. *Staphylococcus aureus* bacteria were stained by a live/dead viability kit, with the dead microorganisms acquiring the red colour, caused by the penetration of the luminescent dye propidium iodide through the damaged cell membrane. Photoluminescence was pumped by 515-nm femtosecond laser pulses with linear (Gaussian beam), circular, azimuthal and radial (Laguerre–Gaussian beam) polarizations, exciting the transverse plasmon resonance of the nanospikes and their apex lightning-rod near-field. According to the numerical electrodynamic modeling, the observed strong increase in the photoluminescence yield for radial polarization, while slightly lower for circular and azimuthal polarizations, compared with the low luminescence intensities for the linear laser polarization, was related to their different laser–nanospike coupling efficiencies.

## 1. Introduction

Surface-enhanced photoluminescence spectroscopy (SEPL) is a promising technique, widely used for the detection of different analytes, as it allows sensing single molecules and bioobjects, as well as trace amounts of inorganic chemical compounds in a non-invasive way. The high efficiency of SEPL is provided by the contribution of electromagnetic and chemical enhancements. An enhancement of the electromagnetic field occurs due to the localized surface plasmon resonance, therefore amplifying the field intensity, while chemical enhancement originates from the charge transfer between the analyte molecule and given nanostructure [1,2]. The wide use of plasmonic nanospikes in spectroscopy applications is mostly due to the local enhancement of the incident electromagnetic field, resulting in the strong plasmon coupling, and therefore enhancing the sensitivity to the trace amounts of chemical compounds or single bioobjects.

Sensors with high efficiency are in need for the detection of hazardous materials trace amounts [3] and bioobjects, including pathogenic microorganisms and a fundamental study of cell structure. Sensors, based on the nanosize plasmonic elements, are profoundly studied for such materials as silver and gold, resulting in the signal enhancement of several orders [3,4].

Plasmonic nanostructures can be fabricated in several ways, including electrodeposition of metals on substrates [3,5,6], nanosphere lithography [7,8] and chemical synthesis of nanoparticles [9]; however, one of the most facile and highly effective ways to fabricate nanostructures with a given morphology is laser ablation, as it allows for the manufacturing of unique nanorelief for a plethora of applications.

Among the different types of structures, nanospikes are of a special interest, as they provide a strong coupling of electromagnetic radiation. Plasmonic metallic nanowires serve as waveguides for the propagation of plasmons, and can localize the electromagnetic energy to subwavelength scales [10]. Plasmonic nanostructures can emit strong fluorescence with excitation at wavelengths in the visible range, which is often contributed to the radiative recombination of Fermi level electrons and sp- or d-band holes [11,12,13]. Gold structures are, therefore, eligible for the fabrication of highly sensitive sensors, as they exhibit larger photoluminescence quantum yields than bulk gold [14], due to the local enhancement of the localized surface plasmon near-field [15], by several orders of magnitude compared with the incident radiation.

The formation of nanospikes on the metal surface (both bulk and deposited as films) was modelled in a number of works [16,17,18,19]. Such nanospikes emerge as a result of nanofoam collapse during spallative ablation by ultrashort (femtosecond) laser pulses [16,19]. The sensor production has to be efficient and low-costing, therefore laser ablation of targets implemented through a high-speed beam moving by using galvanometric scanning heads is one of the most eligible methods [20].

Laser polarization also has a significant effect on the type of the formed nano- and microstructures, as well as on the extent of their excitation during SEPL probing, as they selectively address different types of multipolar Mie resonances [21]. For example, the photoluminescence intensity of R6G dye, when placed as a monolayer on the array of microholes in gold film, was shown to vary depending on laser polarization [22].

In our work, we present the study on the effect of femtosecond laser polarization on the photoluminescence intensity of the red fluorescent dye propidium iodide, which indicates the damaged bacterial membrane, with *S. aureus* as an analyzed bacterial strain. In a great number of works, authors use the resulting sensing devices for the detection of model dyes [10,23] and hazardous chemical compounds [3,4]. The works on bacterial detection are limited to the use of silver nanoparticles [24,25], silicon microcavities [26] or carbon nanodots [27], and do not address the viability of the bacteria. Therefore, we report for the first time the use of gold nanospikes for the combined effect of the bactericidal impact and the detection of the dead cells.

## 2. Materials and Methods

### 2.1. Au Nanospikes Formation

**Figure 1:**
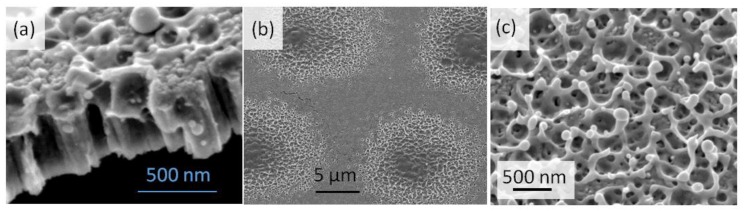
SEM images of the structured Au film: (**a**) side, and (**b**) and (**c**) top view at different magnifications.

Au films with a thickness of ≈500 nm were deposited on silica substrates by magnetron sputtering (SC7620 Mini Sputter Coater, Quorum Technologies) in an argon medium. Nanospikes on Au films were fabricated with the use of a Yb^+^-doped fiber laser Satsuma (Amplitude Systems), with a central wavelength of 1030 nm, pulsewidth of 300 fs and average energy up to 6 µJ. Laser radiation was focused on the Au film surface with the use of an f-theta objective in air, and microcrater arrays were written with the use of a galvanoscanner with an energy of 3 µJ, repetition rate of 2 kHz, scanning speed of 30 mm/s and filling of 75 lines/mm. These parameters were optimal for the formation of equidistant craters without overlapping (Figure 1). Characterization of the surface was implemented by the scanning electron microscope JSM 7001F (JEOL) with an accelerating voltage of 10 keV. For the biofilm analysis, all samples were covered with a 15-nm Au film to avoid the charging of the sample.

### 2.2. Live/Dead Tests

The antibacterial properties of the Au nanospikes were tested on the model strain of *S. aureus*. An 18-h culture was grown in a nutrient medium and then diluted with broth by a 1:100 ratio. After that, all samples were submerged in the Petri dishes, filled with 2 mL of broth culture, and then the bacteria were incubated for 18 h at 37 °C. Biofilm was also grown on the unstructured gold film as a control. A coloration set «Live/Dead Biofilm Viability Kit» was used for the detection of alive and dead bacteria. Fluorescent dyes SYTO^®®^9 (3 μL) and propidium iodide (3 μL) were diluted in 1 mL of distilled water and used to stain alive and damaged bacteria, with propidium iodide (red fluorescent dye) visualizing the bacteria with damaged cell membranes, and SYTO^®®^9 (green fluorescent dye) detecting alive cells. The unstructured gold film and Au nanospikes were stained with fluorescent dyes for 30 min in the darkness and washed with distilled water. Live/dead bacteria were visualized with the use of the fluorescence microscope Nikon H600L with a 40× magnification lens and 600× instrumental magnification.

### 2.3. Sensing of Bacteria

A fresh *S. aureus* strain was placed on the surface of the nano-patterned and unstructured gold film and stained with the fluorescent dye propidium iodide from the «Live/dead Viability Kit». This dye penetrates only the damaged cell membranes, therefore indicating the deceased bacteria. Its photoluminescence takes place at ≈620 nm with the excitation wavelength corresponding to ≈500 nm, which is why laser radiation of 515 nm was used for the testing of the Au nanospikes sensing efficiency.

**Figure 2:**
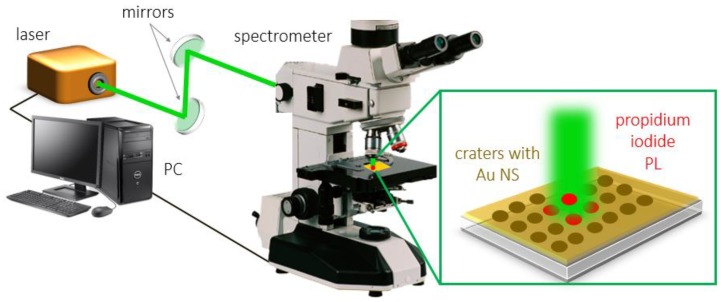
Schematic view of propidium iodide luminescence measurement on Au nanospikes.

Laser pulses (pulsewidth 300 fs) with a low energy (E ≈ 10 nJ, f = 10 kHz) were guided through the back entrance of an upper illumination channel of a microscope–spectrophotometer MSFU-K (LOMO, 350–900 nm, 0.2 nm resolution, slit 0.3 mm) through the 50% beam splitter and were focused onto the patterned gold film in an air medium through the microscope objective with a numerical aperture NA of 0.65 (magnification 40×) (Figure 2) into spots with diameters of 25 µm. Initially, a Gaussian beam was transformed into the donut-shaped (Laguerre–Gaussian) one with the use of a commercial S-waveplate (Altechna R&D, Vilnus, Lithuania) converter. The PL spectra of propidium iodide, indicating the deceased bacteria, were acquired for the different laser polarizations, changed by an S-waveplate.

Photographs of the surface, irradiated with a 515-nm laser beam, were taken with the use of a CCD (charge-coupled device) camera in order to illustrate the fluorescence of propidium iodide.

The resulting signal was acquired from an area of one microcrater.

### 2.4. Numerical Simulation

In this work, we carried out a numerical simulation based on a finite-difference time-domain (FDTD) scheme to characterize the electric field interaction with the Au nanospikes. We used either a Gaussian beam with linear polarization or a donut-shaped beam with azimuthal or radial polarization for illuminating an array of nanospikes on a semi-infinite silica glass substrate. The height of the nanospike was 250 nm, the nanospike base radius was 35 nm, the pinnacle radius was 50 nm, the period was 300 nm and the substrate side length was 900 nm. A perfectly matched layer (PML) boundary condition was applied for the computational domain.

## 3. Results

### 3.1. Antibacterial Activity

**Figure 3:**
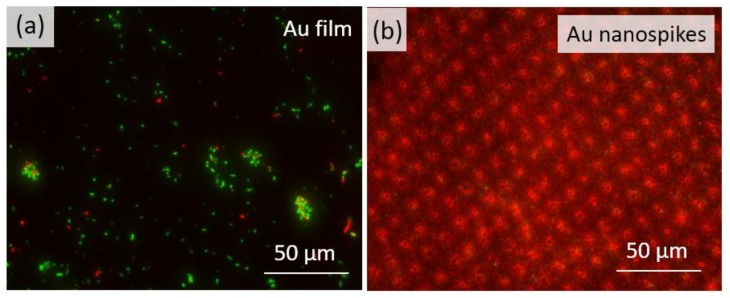
Fluorescent microscope photographs of alive (green) and dead (red) *S. aureus* bacteria on the unstructured Au film (**a**) and Au nanospikes (**b**).

The resulting fluorescent microscope photographs are shown in Figure 3. Red stains indicate the effective death of *S. aureus* bacteria on the Au nanospikes.

**Figure 4:**
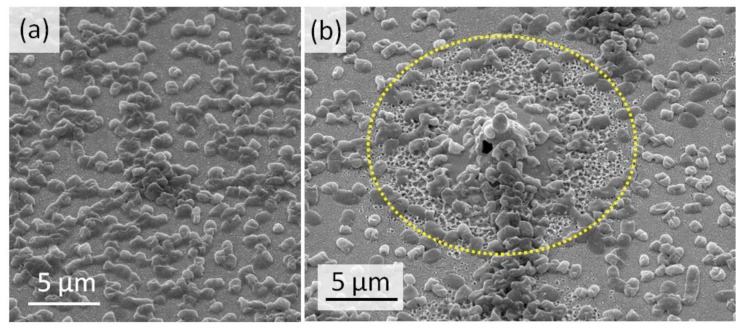
SEM images of *S. aureus* bacteria on the unstructured Au film (**a**) and on Au nanospikes (**b**).

The SEM characterization shows the fewer populations of bacteria on the nanostructured Au surface (Figure 4a,b), with a ruptured membrane of the bacteria and their further deformation. The Au nanospikes mainly consist of ≈300-nm high stems with ≈100–200 nm droplets frozen atop of them (Figure 1c). The observed relief corresponds to the morphology, often seen after spallative ablation [16,17].

### 3.2. PL Sensing of Bacteria

**Figure 5:**
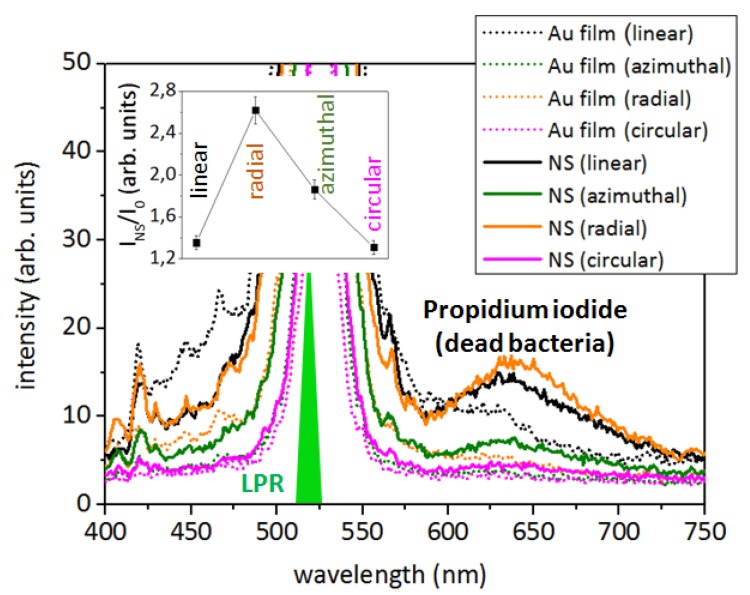
Photoluminescence spectra of the fluorescent dye propidium iodide, indicating the dead bacteria (broad peak at 620 nm) on the surface of the unstructured Au film and nanospikes (NS). The expected plasmon resonance (localized plasmon resonance, LPR) of the Au structures corresponds to 520 nm. Inset: normalized intensity of the propidium iodide peak at linear, radial, azimuthal and circular polarizations.

The photoluminescence spectra of the dead *S. aureus* bacteria with excitation of the Au nanospikes by linear, radial, azimuthal and circular polarizations are presented in Figure 5. It is known, that propidium iodide in aqueous solutions has an excitation maximum at ≈490 nm and an emission maximum at 640 nm. When it is bound to DNA, its excitation maximum shifts to the red and the emission maximum shifts ~15 nm to the blue, therefore resulting in an excitation maximum at 535 nm and an emission maximum at ≈620 nm [28,29].

**Figure 6:**
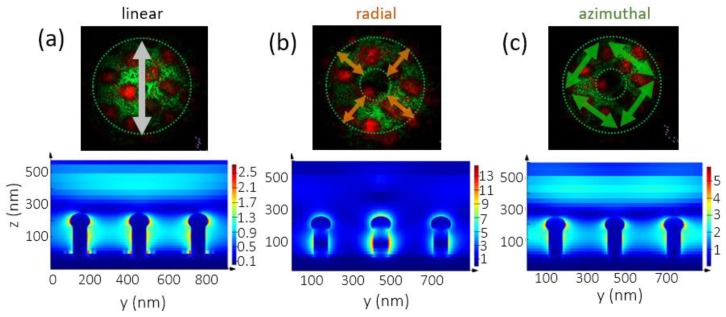
Numerical simulation of the electric field distribution for laser radiation with λ = 600 nm for the different polarizations: linear (**a**), radial (**b**) and azimuthal (**c**). Color scale in |E|/|E_0_|.

**Figure 7:**
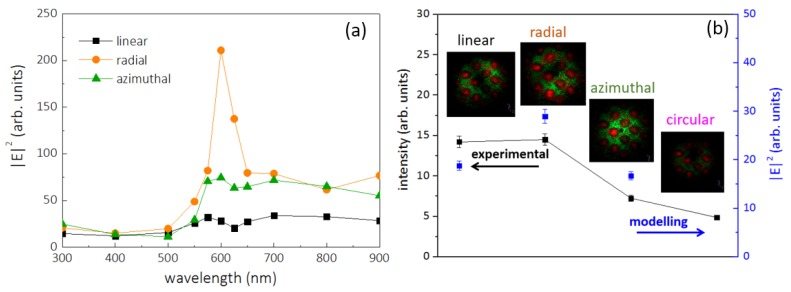
(**a**) Calculated maximal value of |E|^2^ for the different polarizations depending on the wavelength. (**b**) Dynamics of the PL intensity at 625 nm versus different laser polarizations (left axis) and the corresponding simulation results of |E|^2^ measured at 515 nm.

The signal with maximal intensity was acquired with radial laser polarization. The red spots on the microphotographs (Figure 6, upper line; Figure 7, insets) correspond to the microcraters on the Au film surface, which are filled with nanospikes, and the green area around them is the excitation laser beam.

The visualization of the numerically calculated electric field distribution is shown in Figure 6, on the lower line. The experimentally observed nanomorphology was approximated to the combination of nanopillars with a round cap on top (Figure 1c). The electric field maximum is located around the pinnacle of each nanospike for beams with linear and azimuthal polarizations. On the contrary, for the case of radial polarization, only the central nanospike of the array demonstrates the strong electric field enhancement. Moreover, the maximum is located closer to the base of the spike.

The maximal value of |E|^2^ was calculated versus the laser pump wavelength, which allowed the revealing of the strongest coupling of radially polarized laser radiation with the Au nanospikes at ≈625 nm (Figure 7a). The simulation results are in a good qualitative agreement with the experimental ones (Figure 7b). However, in the real case, at high-NA focusing, the radially polarized light produces a rather strong longitudinal component [30], which additionally drives on the spikes a strong “lightning-rod” effect [31], thus enhancing the resulting photoluminescence yield [10,22,32].

## 4. Discussion

The nanospikes formation is mainly attributed to the material redeposition during the spallative ablation regime. The energy used in our work reaches 3 µJ, with the laser fluence corresponding to ≈1.5 J/cm^2^, which is higher than the threshold fluence for spallation of Au in air [33].

**Figure 8:**
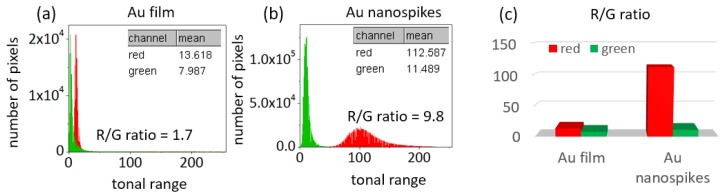
(**a**, **b**)—color histograms, acquired from the microphotographs in Figure 3; (**c**)—red to green (R/G) ratios of photographs with the Au film and Au nanospikes.

Red propidium iodide dye penetrates the damaged cell membrane, therefore indicating dead bacteria, and green SYTO9 dye indicates alive microorganisms (Figure 3 and Figure 6). Therefore, through the analysis of fluorescent microscope photographs, one can define the antibacterial efficiency by measuring the red and green signals on the RGB color histogram of the resulting microphotographs. The data obtained with the use of the ImageJ software are presented in Figure 8a,b, with the X-axis corresponding to tonal range and the Y-axis indicating the number of pixels in the photograph.

**Figure 9:**
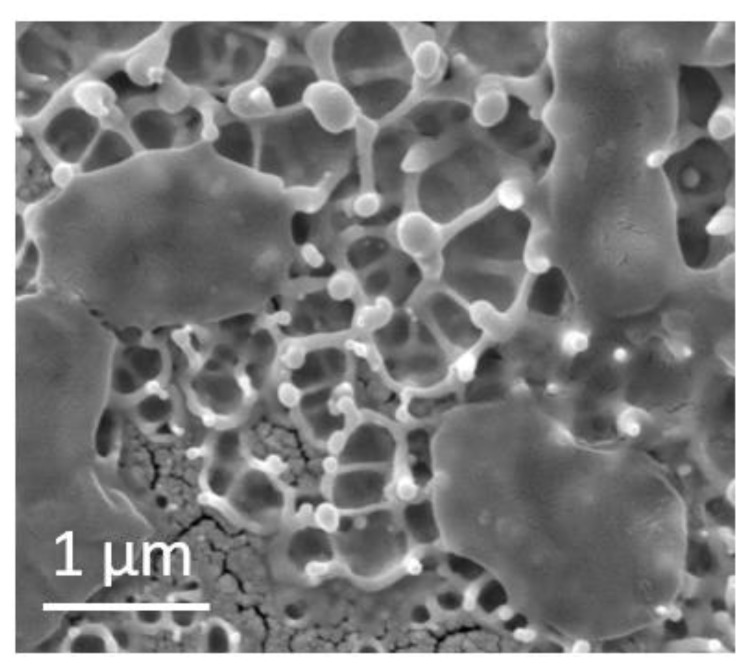
SEM image of damaged S. *aureus* cells on the surface of Au nanospikes.

As a result, the Au nanospikes show a high antibacterial efficiency against *S. aureus*, which is caused by the mechanical rupture of the bacterial membrane on the sharp Au nanospikes. The mean size of *S. aureus* bacteria is 1 µm, which covers the area of approximately 7–10 Au nanospikes and ensures the cell death (Figure 9).

**Figure 10:**
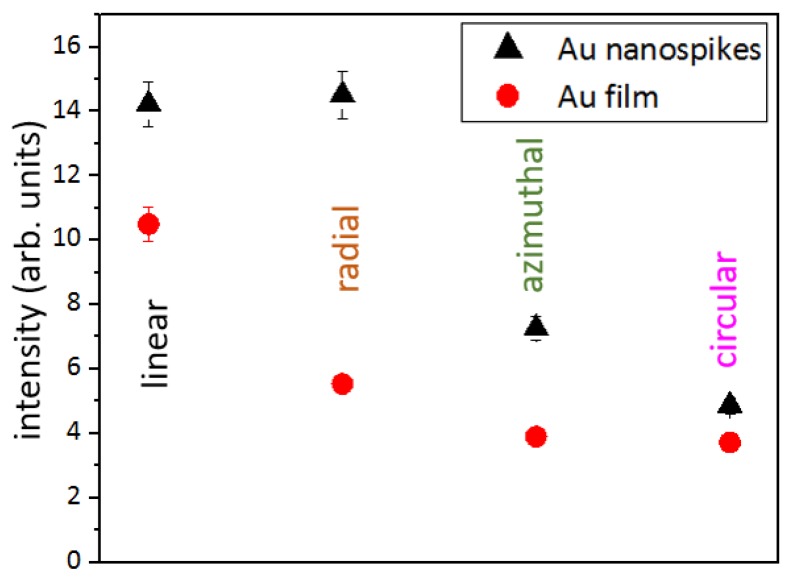
PL intensity measured at 625 nm for the unstructured Au film (red circles) and Au nanospikes (black triangles) for linear, radial, azimuthal and circular polarizations.

In our photoluminescence measurements, the Au samples were irradiated at the 515-nm wavelength, which lies close to the plasmon resonance in gold [34]. Such spectra, acquired from the unstructured Au film, exhibit a low luminescence intensity of propidium iodide at radial, azimuthal and circular polarizations (Figure 5 and Figure 10). Nanospikes enhance the PL intensity for these polarizations two-fold (three-fold for radial polarization) (Figure 10).

The laser beam was focused onto one crater, which was filled with Au nanospikes, and therefore the coupling of the laser radiation occurred only to the structured surface.

It was shown previously, that even on an unstructured Au film, the excitation of analyte is maximal with the use of radial polarization [32]. This was attributed to the excitation of plasmons in Au nanocrystallites both in the film plane and in the perpendicular direction due to the appearance of the longitudinal component of the electric field with radial polarization.

## 5. Conclusions

In this work, we studied the detection of live/dead *Staphylococcus aureus* bacteria with the use of sharp golden nanospikes, fabricated by 1030-nm 300-fs laser pulses. The Au nanospikes were tested as potential elements for antibacterial surfaces and plasmonic luminescence sensors. Photoluminescence of the red dye, indicating the cell death (propidium iodide) was pumped by 515-nm femtosecond laser pulses with linear (Gaussian beam), circular, azimuthal and radial (Laguerre–Gaussian beam) polarizations, exciting the transverse plasmon resonance of the nanospikes and their apex lightning-rod near-field. The highest signal enhancement (three-fold) was acquired with radial polarization, which was attributed to the appearance of the longitudinal component of the electric field during the laser–nanospike coupling.

## 6. Patents

There are no patents resulting from the work reported in this manuscript.
